# Early detection of potential errors during patient treatment planning

**DOI:** 10.1002/acm2.12388

**Published:** 2018-07-05

**Authors:** Danielle Lack, Jian Liang, Lisa Benedetti, Cory Knill, Di Yan

**Affiliations:** ^1^ Department of Radiation Oncology Beaumont Health System – Troy Troy MI USA; ^2^ Department of Radiation Oncology Beaumont Health System – Royal Oak Royal Oak MI USA; ^3^ Department of Radiation Oncology Beaumont Health System – Dearborn Dearborn MI USA

**Keywords:** automation, plan data communication, quality control, treatment planning

## Abstract

**Purpose:**

Data errors caught late in treatment planning require time to correct, resulting in delays up to 1 week. In this work, we identify causes of data errors in treatment planning and develop a software tool that detects them early in the planning workflow.

**Methods:**

Two categories of errors were studied: data transfer errors and TPS errors. Using root cause analysis, the causes of these errors were determined. This information was incorporated into a software tool which uses ODBC‐SQL service to access TPS's Postgres and Mosaiq MSSQL databases for our clinic. The tool then uses a read‐only FTP service to scan the TPS unix file system for errors. Detected errors are reviewed by a physicist. Once confirmed, clinicians are notified to correct the error and educated to prevent errors in the future. Time‐cost analysis was performed to estimate the time savings of implementing this software clinically.

**Results:**

The main errors identified were incorrect patient entry, missing image slice, and incorrect DICOM tag for data transfer errors and incorrect CT‐density table application, incorrect image as reference CT, and secondary image imported to incorrect patient for TPS errors. The software has been running automatically since 2015. In 2016, 84 errors were detected with the most frequent errors being incorrect patient entry (35), incorrect CT‐density table (17), and missing image slice (16). After clinical interventions to our planning workflow, the number of errors in 2017 decreased to 44. Time savings in 2016 with the software is estimated to be 795 h. This is attributed to catching errors early and eliminating the need to replan cases.

**Conclusions:**

New QA software detects errors during planning, improving the accuracy and efficiency of the planning process. This important QA tool focused our efforts on the data communication processes in our planning workflow that need the most improvement.

## 1 INTRODUCTION

Physics pretreatment plan review has been shown to be one of the most effective ways to reduce errors in radiotherapy.^1^ During this review, the physicist will independently evaluate the plan quality based on clinical goals, as well as verify that all of the technical details of the plan are correct. This process has become more challenging for the physicist to perform in the modern radiotherapy era for a number of reasons. First, the number of treatment methods being offered along with their complexity continues to grow.^2^ In fact, it was estimated in a 2009 study that progressing from consult to treatment delivery for an external beam radiotherapy patient required approximately 270 different steps. Of those 270 steps, it appears that around 100 are attributed to the treatment planning process.^3^ Secondly, the number of computer systems required in a standard radiotherapy treatment has also grown. In a 2013 article by Moore et al.,^4^ they note that there are at least 11 different “software functionalities” involved in a standard radiation therapy clinic. This means that in order to complete a pretreatment physics review, the physicist must navigate through multiple electronic workspaces as well as check a significant number of parameters, often manually which can be quite complicated.

The increasing demands on physics resources to perform independent plan review have been recognized by the radiation oncology community. In response, several investigators have developed and implemented software programs which automate portions of the physics pretreatment check. These programs check items such as patient setup and prescription information, beam parameters, dose computation settings, optimization parameters, and dosimetric goals.^5^, ^6^, ^7^, ^8^, ^9^, ^10^, ^11^, ^12^, ^13^, ^14^, ^15^, ^16^, ^17^ However, one area of the treatment planning process that has not been the focus of these software programs is checking the integrity of data communication processes between various software and hardware platforms. For example, a patient may be CT simulated and at the time of simulation, the therapist may accidentally mistype the patient medical record number (MRN) into the CT software. Images are acquired and imported into the treatment planning system and a plan is created and approved. During export of the DICOM plan and CT information, the dosimetrist discovers the error in MRN as the plan and CT information are not able to be transferred to the correct patient in the record and verify system. This means that the patient cannot be treated unless the error is fixed, requiring that several steps in the planning process be repeated a second time. While this type of data error may not result in mistreatment of the patient, it is costly in terms of the efficiency of plan creation, since it takes significant time to find the error and repeat necessary planning steps, resulting in delayed start times for patients.

In this work, we investigate the causes of various data errors that can occur during the treatment planning process and develop a software tool that can automatically detect them in advance. The impact of this software on the accuracy and efficiency of treatment plan creation at our center is also presented.

## 2 MATERIALS AND METHODS

### 2.A Health system data environment

Our health system data environment is quite complex consisting of three clinical Departments, five CT simulators (Brilliance CT Big Bore, Philips Healthcare, Bothell, WA, USA; SOMATOM Sensation CT, Siemens Healthineers USA, Malvern, PA, USA), two separate record and verify system databases (Mosaiq, Elekta, Stockholm, Sweden), a centralized treatment planning system (Pinnacle v.14.0, Philips Healthcare, Bothell, QA, USA) and 10 linear accelerators (Elekta, Stockholm, Sweden) with OBI software (XVI v.5.0, Elekta, Stockholm, Sweden).

### 2.B Identification of data errors

Between the years of 2012–2015, data errors that were caught clinically in our Department either by the dosimetrist at the time of planning, physicist at the time of pretreatment review, or the therapist during treatment were tabulated. A review of the data errors found that they could be classified into two major categories: (a) data transfer errors and (b) user errors in the treatment planning system. For each of these data errors, a root cause analysis (RCA) was performed. This type of procedure is a well‐established technique to manage errors in healthcare and involves starting at the clinical incident where the error was discovered and tracing backwards through each step of the treatment workflow until a root cause is identified.^2^, ^18^, ^19^ RCA analysis was based on a review of pertinent files and databases related to each of the data errors.

### 2.C Data error tracking software development and implementation

Once the root causes of the data error subset were determined, a software tool to automatically monitor related files and databases for potential errors was developed. In our clinical setting, we use a unix‐based treatment planning system and a windows‐based record and verify system. As such, a windows‐based software tool could be easily implemented without the need to install additional software on the treatment planning system servers. The software tool was developed using the C/C++ language on a windows platform. It uses the PostgresSQL open database connectivity (ODBC) driver to access our centralized Pinnacle treatment planning system patient Postgres database, and uses the SQL Server ODBC driver to access our two windows‐based Mosaiq MSSQL record and verify system databases. The software uses a windows file transfer protocol (FTP) service (mainly, the CInternetSession::GetFtpConnection function), to scan the treatment planning system's internal files. In order to ensure that the software had read‐only access to these files and could not accidently modify any plan data, a special interface was developed using the C/C++ programming language. Details regarding which file type (image file, TPS internal file etc.) as well as what information within each file is needed for the software to check for a given data error is detailed in Table 1.

**Table 1:** Summary of data errors and root causes identified through root cause analysis procedure. Details on files scanned and information needed to check for a given error is summarized in the rightmost column

Error type	Root errors detected	Cause(s)	Significance	How software detects data error
Data transfer errors	Image slice missing from dataset	DICOM transfer and image reconstruction error between TPS and PACS/CT software	Incomplete generation of target/normal tissue contours	Check **CouchPos** inside the image files: ImageSet_*.ImageInfo
	Wrong patient identification entered	Manual entry of patient information incorrect	Prevents transfer of CT/plan data between TPS, R&V, and OBI software	Check patient identification inside Pinnacle's database table *patient*, Pinnacle internal file *Patient*, DICOM tag in the planning CT image, Mosaiq database *Ident* table and *vw_Patient* view, and compare the common fields
	Wrong image DICOM tag generated	Bug in CT software creates wrong DICOM tag	Prevents transfer of CT data between R&V and OBI software	Check **SeriesInstanceUID (0020, 000e)** in image DICOM files
TPS user errors	Incorrect CT selected as planning CT	Diagnostic reference CT manually selected as planning CT	Incorrect patient setup and CT‐density table applied	Check **CT Station name (0008,1010)** in image DICOM files and ImageSet_*.header. If the station name is not one of the Department CT machines, the error message will be sent out
	Incorrect CT‐density curve applied	TPS software/dosimetrist selects wrong curve	Dosimetric discrepancies of 1%–3% in target coverage	From DICOM image file, obtain **CT Station name** and **KVP (0018,0060).** From *plan.Trial* find CT‐density table, then verify the CT‐density table using rules from our Department policy
	Incorrect patient image in secondary image list	User imports image to wrong patient	Incorrect reference patient dataset used for planning	Compare the **patient name** and **MRN information** obtained from the planning CT and secondary images. TPS database table name is *imageset*
Other	Other errors (mostly R&V performance errors)	R&V service not working and needs reset	DICOM data conversion from TPS to R&V system stopped, R&V image review slow	Monitor the incomplete events from Mosaiq database table *WorkQueueElement*

TPS, treatment planning system; R&V, record and verify system; OBI, on‐board imaging system. Keywords to be searched in TPS internal file and database are highlighted as bold font type.

The software program was created by focusing on one data error at a time, for example, data error X. During the development phase, patients from the RCA that had data error X were used to test the software. If a false negative was encountered, then the software was modified and the process repeated until data error X could reliably be detected without false negatives. Once this was completed, verification was performed by creating data error X for a new set of patients and running the software. If false negatives were encountered, the software was modified further and testing was performed again. This iterative process of modifying and testing the software was repeated until it was confirmed that the software could correctly identify data error X without false negatives.

The data error tracking tool was implemented in our clinic at the end of 2015 and has been fully operational since the beginning of 2016. Since then there have been no false negatives discovered by the software. All false positives have been tied to research or quality assurance (QA) patient entries created in the Pinnacle database without a corresponding entry in the record and verify system databases. To minimize the false positives detected by the software for research or QA cases, some rules have been built into the software to ignore an entry if it has “QA” or “TEST” in the name and staff members have been notified to include these keywords when creating their test patients.

**Figure 1:**
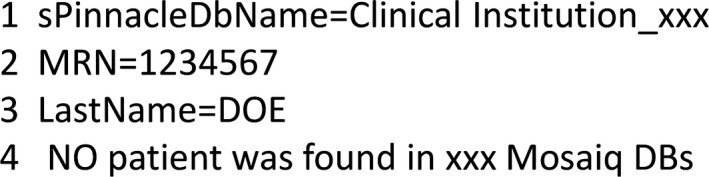
Sample data error report generated by software tracking tool. In this example, the MRN for the patient on the helical CT is mismatched with the Mosaiq database. This is because the CT technician typed in a wrong MRN.

Currently, the software tool is run every evening and will scan all database files modified since the previous scan the night before. Whenever an error is detected a report is generated locally on the computer that runs the software and a review request with a short summary of the error (without patient identification information) is emailed to a physicist. The physicist then accesses the full data error report from the local computer and investigates the error. A sample data error report from the local computer is shown in Fig. 1 for a patient whose MRN on the reference DICOM CT is mismatched with the MRN in the Mosaiq database. Once the physicist confirms the error is true, the responsible clinicians (the CT technicians and dosimetrist for this case) are notified to correct the error in order to prevent it from propagating further in the treatment planning chain.

### 2.D Time‐cost analysis of data errors

In our experience, data errors detected in the later stages of the planning process have required time by the physicist to analyze and correct, which can significantly delay the start of treatment for patients. In an effort to characterize the improvements in clinical efficiency as a result of implementing this software tool, a time‐cost analysis was performed. For each data error type, the maximum time necessary to identify and correct the error with and without the software implemented was estimated. Time estimates were based on our clinical experience. The difference in these time estimates for each error was then used to calculate the maximum potential delay in the start of treatment for patients without the software implemented.

## 3 RESULTS

### 3.A Root cause analysis of data errors

A summary of data errors as well as their root causes as identified through the root cause analysis process is also presented in Table 1.

#### 3.A.1 Data transfer errors

There were three main data transfer errors identified in our study. The first was an image slice missing from an imaging dataset. This error can be caused by (a) a DICOM transfer error between the CT console and the treatment planning system, (b) an error in the reconstruction of the 4D average image in the CT software, or (c) a Pinnacle treatment planning system import error for MR images. Specifically, oblique MR images must be converted by the dosimetrist prior to being imported to the treatment planning system. In our clinic, this conversion process is done using the MIM software platform for fusion and image registration (MIM software Inc., Cleveland, OH, USA). This converts the MRI to the planning CT imaging coordinate system, after which images can be imported into the treatment planning system. If this conversion process is not done, then the display of the images in the treatment planning system can be incorrect.^20^ If the display is incorrect, then the treatment planning system records a variable slice thickness for each MR image in the dataset. Since the image could be misrepresented in the planning system, this scenario was classified under the category of image slice missing from a dataset. The clinical significance of this type of error is that contours for target volumes and/or normal tissue structures located in the region of missing imaging data are incomplete or misrepresented. These incomplete contours can cause a failure to export the structure set from the planning system to the record and verify system as well as the on‐board imaging system at the end of the treatment planning process. Depending on the exact cause, this error can be remediated by (a) re‐exporting the CT from the CT console, (b) reconstructing the 4D average image a second time and re‐exporting the images, or (c) properly doing an MRI conversion for oblique images in the MIM software, all followed by reimporting the images into the treatment planning system. If contours and planning were completed prior to the error being found, then both are copied from the previous image set to the new image set and the dose is recalculated. Once corrected, the clinician will need to review the images, target, and organs at risk contours as well as the recalculated plan. Depending on where the missing imaging slice is located (i.e., in target volume or critical organ at risk), replanning of the case may be necessary.

The second data transfer error identified was wrong patient information entry caused by an incorrect manual entry of patient information either in the CT simulation software or treatment planning system. If the error is in the MRN, the reference CT and DICOM plan data cannot be sent from the treatment planning system to the correct patient in the record and verify system. If the error is in the patient gender, data can be transferred successfully to the record and verify system, but whether or not it can be sent correctly to the on‐board imaging software depends on how the gender mismatch occurred. Patient gender information is stored in two places in the TPS: the CT DICOM tag and the Pinnacle Patient Information file. If the gender located in these two files match, but are different from Mosaiq, then data can be transferred successfully to both the record and verify system and OBI software. However, if the gender differs between these two files, then the reference CT, DICOM plan and structure set are not associated with one patient entry in the XVI database, but instead are separated into two patient entries based on gender. In this case, the reference CT may be listed under “John Doe” patient entry 1 while the DICOM structure set information shows up under “John Doe” patient entry 2. DICOM plan information may fall in either entry 1 or 2 depending on which entry matches the gender information recorded in Mosiaq. Since the DICOM images, plan, and structure set are not all associated under the same patient entry, the data cannot be imported to create the XVI reference dataset and image guidance with cone‐beam CT is prohibited.

Manually determining the cause of the dataset mismatch can take significant time to identify and correct. If the misinformation is tied to the reference CT, then for data consistency purposes, it should be corrected at the CT console, reimported to the treatment planning system, and then resent to the record and verify system. Additionally, if an erroneous reference CT was successfully sent to the record and verify system, this CT image as well as any associated patient setup data has to be removed prior to re‐export of the new CT to the record and verify system. It should be noted that if the gender for the CT DICOM tag and Pinnacle Patient information match, but are different from Mosaiq, it is still our practice to fix the gender mismatch. This is to prevent any issues with gender in the future if the patient has to return for subsequent radiation treatment.

The third data transfer error reviewed was an incorrectly generated image DICOM tag. This error was associated with a bug in the CT simulation software for two of our CT simulators which were creating two consecutive periods in the DICOM tag in the reconstructed average image of 4DCT datasets. With an incorrect DICOM tag in place, the record and verify system is able to receive and store the reference image, but the reference CT cannot be transferred from the record and verify system to the on‐board imaging system. To fix this problem, the image tag must be corrected and the image set must be reimported into the treatment planning system and then resent to the record and verify system. In this case, the erroneous reference image in the record and verify system cannot be deleted by clinical users. To remove the image, help from vendor technical support is required, which from our experience can take multiple days to complete.

#### 3.A.2 Treatment planning system errors

Three main TPS errors were identified in our analysis. The first was an incorrect CT dataset selected as the planning CT. This error is caused by an incorrect manual selection of a CT dataset acquired on an unknown CT device as the reference planning CT. An unknown CT device is any device outside of our five Department CT units and for which CT scanner information is unavailable in the treatment planning system database. The significance of this error is that the patient setup used for planning as well as the CT‐density curve applied in the treatment planning system for dose calculation are incorrect. To fix this, the correct reference CT must be selected for treatment planning, and depending on how different the patient positioning and anatomy is between the two datasets, significant work may be needed to recontour and replan the case.

**Figure 2:**
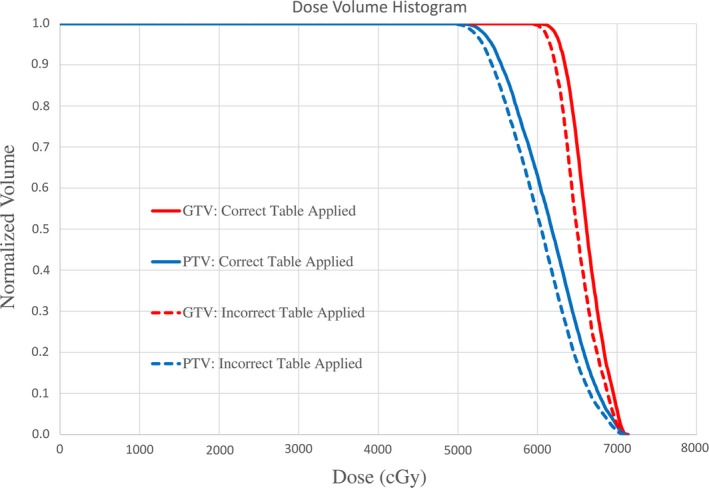
Example of the dosimetric impact of applying an incorrect CT‐density curve for dose calculation. PTV (blue) and GTV (red) DVH curves are shown for a lung SBRT patient. Discrepancies in D95 target coverage with the correct 120 kVp curve applied (solid lines) and incorrect curve applied (dashed lines) is ~3%.

The second treatment planning system error identified was an incorrect CT‐density curve applied to the reference CT for dose calculation. This is caused by the treatment planning system software or dosimetrist selecting the wrong curve. Our CT scanner database is quite complex due to the number of CT simulators we have in our Department (five in total between three sites) as well as the fact that our database also includes older CT‐density curves to allow for dose calculation from previous versions of our treatment planning system. Keeping older CT‐density curves in the database allows for the generation of composite dose distributions for patients with previous treatment. Our treatment planning system will not allow for a reference CT to be imported without selecting a CT‐density table for dose calculation. Therefore, it has been configured to automatically select the correct CT‐density table for dose calculation based on the CT KVP value, manufacturer name and model information in the DICOM tag. Unfortunately, when 4DCT average images are reconstructed at the CT console, the CT model information is absent. Lack of this scanner information results in the treatment planning system selecting a default CT‐density table for the plan, which may be incorrect. Clinically, the application of an incorrect CT‐density table can result in dosimetric discrepancies of 1–3% in target coverage as highlighted by the DVH plot for a lung SBRT patient in Fig. 2. For this patient, DVHs for the GTV and PTV are shown. The solid lines represent the calculated DVH with the correct 120 kVp CT‐density table applied. The dashed lines represent the calculated DVH with an incorrect CT‐density table applied. The difference in the D95 between the two curves for both the GTV and PTV is ~3%. To fix this error, the correct CT‐density table must be applied and depending on the dosimetric difference in dose calculation, adjustments to the treatment plan may be required.

The last treatment planning system error reviewed was an incorrect patient image in the secondary image list. This is caused by the dosimetrist manually importing a secondary image into the wrong patient. If the image set that is incorrectly imported is for a similar treatment site, for example, both images are of the pelvis, then this type of error may not be caught unless the clinician carefully verifies image information such as the image name displayed in the corner of the image view window. Since these secondary images are generally fused to the reference CT, this means that incorrect patient image information/anatomy is being used to guide contouring and treatment planning. To fix this, the incorrect dataset must be removed and if that dataset was mistakenly used to guide treatment planning, replanning is necessary.

#### 3.A.3 Other errors

The last category in Table 1 specifies other errors that were investigated. These errors did not occur often in the clinic, but were easy to incorporate into the software tool for monitoring. For example, performance errors within our record and verify system, specifically the Mosaiq Work Queue Element (WQE) Processor service were monitored. This service activity is responsible for data conversion within the record and verify system, and runs on the record and verify system server that does not reside in our Department. When this service stops working, DICOM data transfer to the record and verify system gets backlogged as data are received by the record and verify system's import filter, but cannot be assigned for further processing. For instance, DRR images are sent from Pinnacle to the record and verify system, but are unable to be assigned to the corresponding patient. Additionally, this backlog of patient data results in slow performance of image review activities in the record and verify system (e.g., CBCT image review). To catch this error, our software tool continually monitors the WQE service activities to see if there are an unexpectedly high number of incomplete jobs in the record and verify system database. Once the error is detected, the hospital information technology department must be contacted to reset this service on the server computers.

### 3.B Frequency of data errors detected

The data error tracking tool was implemented in our clinic at the end of 2015 and was fully operational for the 2016 year. In that year, 79 data errors and 5 performance errors with the record and verify system were detected by our tool. Using the number of simulations performed in 2016 as a surrogate for the total number of patient plans generated in that year, this corresponds to a data error frequency rate in our clinic of ~2.3%. The breakdown of errors by type and frequency is presented in Table 2, which shows that the most frequent data errors detected were: wrong patient identification entered (35 occurrences), incorrect CT‐density curve applied (17 occurrences), and image slice missing from dataset (16 occurrences).

**Table 2:** Data error tool tracking results for 2016 and 2017 broken down by root error type. Performance errors by the record and verify system were not classified as a true “data” error

Root errors detected	2016 Frequency	2017 Frequency
Image slice missing from dataset	16	3
Wrong patient identification entered	35	36
Wrong image DICOM tag generated	7	0
Incorrect CT selected as planning CT	3	1
Incorrect CT‐density curve applied	17	2
Incorrect patient image in secondary image list	1	0
Other errors (mostly R&V performance errors)	5	2
Total data errors detected	79	42
Total errors detected	84	44

### 3.C Clinical interventions as a result of software

Based on the 2016 data error results, two main actions were implemented into our clinic to improve our data error rate. First, clinicians were educated on the cause and consequence of data errors they were responsible for. This increased awareness in the clinic of what data errors were being seen in our treatment planning workflow and how they could be prevented. Secondly, improvements were made to some of the data communication procedures used in our treatment planning workflow. For example, one of the main data transfer errors identified was an incorrect DICOM tag being generated as a result of a bug in the software for two of our CT simulators. To prevent this error from propagating in the treatment planning workflow, a script was written to remove the consecutive period in the DICOM tag file. This script is currently run by a physicist prior to importing any 4DCT average images that have been generated on these two CT simulators into the treatment planning system.

With these actions implemented, it was interesting to see what the impact was on our clinical data error rate in 2017. In total, 42 data errors and 2 record and verify system performance errors were detected, corresponding to a data error rate of ~1.1% based on the total number of simulations performed that year. This demonstrates that the clinical interventions implemented effectively cut our data error rate in half. The breakdown of data errors by type and frequency for 2017 as compared to 2016 is also presented in Table 2. Interestingly, as a result of the clinical interventions implemented, data transfer errors that were software based, such as the wrong DICOM image tag being generated, decreased significantly from seven occurrences in 2016 to none in 2017. On the other hand, the frequency of manual data entry errors, such as a wrong patient identification error, did not change with 35 errors detected in 2016 and 36 errors detected in 2017.

### 3.D Time‐cost analysis of data errors

Table 3 presents the maximum time estimates to fix a given data error with and without the software tool implemented. Prior to the software being implemented, these data errors were discovered at the end of the treatment planning workflow after the plan had already been completed and exported to the record and verify system. In this worst‐case scenario, significant time is needed to replan the case and for some data errors, work with the vendor to remove erroneous images from the patient chart. With the software running daily, errors are caught at the beginning of the workflow, often before any contouring has begun. Estimates without the software are based on the worst‐case scenario and take into account the time needed for replanning and or vendor support.

**Table 3:** Maximum time estimates to correct a given data error with and without the software tool implemented. One day was considered to be equivalent to an 8‐h work day

Root Error	Time to fix with software tool implemented	Maximum time to fix without software tool implemented
Image slice missing from dataset	30 min	1 day
Wrong patient identification entered	10 min	1 day
Wrong image DICOM tag generated	10 min	1 week
Incorrect CT selected as planning CT	10 min	2 days
Incorrect CT‐density curve applied	5 min	4 h
Incorrect patient image in secondary image list	10 min	1 day

Using the time estimates from Table 3, the maximum time cost per year to correct the data errors detected in 2016 was calculated and results are presented in Table 4. For calculation purposes, 1 day was considered equivalent to 8 working hours and 1 week was considered equivalent to 40 working hours. Without the software tool implemented, we estimate a maximum delay in start times for patients of ~812 h in 2016. Comparatively, with the software tool implemented, we estimate that the delay in patient start times would be reduced to ~17 h, demonstrating a maximum potential time savings of ~795 h that year. When a similar analysis was performed for the 2017 data errors, it was estimated that the total time cost to correct errors with the software implemented was further reduced to ~8 h due to the reduced number of errors that occurred that year.

**Table 4:** Time‐cost estimate in hours to correct data errors detected with and without the software tool implemented in 2016 and with the tool implemented in 2017

Root errors detected	Time cost to correct error without software 2016	Time cost to correct error with software 2016	Time cost to correct error with software 2017
Image slice missing from dataset	128.0	8.0	1.5
Wrong patient identification entered	280.0	6.0	6.0
Wrong image DICOM tag generated	280.0	1.2	0.0
Incorrect CT selected as planning CT	48.0	0.5	0.2
Incorrect CT‐density curve applied	68.0	1.4	0.2
Incorrect patient image in secondary image list	8.0	0.2	0.0
Total	812.0	17.3	7.9

## 4 DISCUSSION

The increased complexity of creating and delivering a radiation therapy treatment plan today has resulted in the medical physics community taking a step back and re‐evaluating how quality management is executed in radiation oncology. Historically, quality management has been based on device‐specific quality assurance measures where every device involved in patient treatment is tested at various frequencies and held to specific tolerances. This approach is time intensive for the medical physicist who already faces issues with limited resources. Furthermore, the problem with this approach as highlighted in the recent TG‐100 report, is that this technique fails to catch errors that are tied to problems with the clinical process itself.^2^

In this work, we took a closer look at data errors in the treatment planning process at our institution. Through performing a root cause analysis of data errors that occurred in our clinic we gained a better understanding of the interactions between human users and individual devices in our treatment planning workflow and the impact on treatment outcomes. Interestingly, a significant proportion of the data errors that were identified are tied to manual errors by humans (35 of 79 errors in 2016 and 36 of 42 errors in 2017). These range from typographical errors of patient demographics in the CT simulation software or treatment planning system to incorrect manual selection of the CT dataset that is to be used for planning. The dosimetric consequences to the patient for some of these errors such as incorrect CT used for planning can be significant. However, in general, the main consequence of these errors as shown in Table 1 is an inability to treat the patient due to rejection of DICOM data by a treatment device/software that comes later in the treatment chain. This means patient start times are delayed and inefficiencies in the treatment planning process are introduced due to the time it takes to find the data error and repeat treatment planning steps a second time.

As a result of the understanding gained from this work, some interventions were introduced into our clinical workflow in an attempt to reduce the data error rate. For example, once it was understood that two of our CT simulators had a software bug that was creating an incorrect DICOM tag for 4DCT images, a script was written to fix the DICOM tag. This script is now run prior to importing any 4DCT average image from those two simulators into the treatment planning system. This intervention reduced the frequency of this data error from seven occurrences in 2016 to none occurrences in 2017. Similarly, once it was understood that CT scanner model information was missing in the DICOM tag file for 4DCT average images, a script was written in Pinnacle which is run by the dosimetrist at the start of planning to check if the CT‐density curve applied is correct. This reduced the frequency of this data error from 17 occurrences in 2016 to 2 occurrences in 2017. Manual data errors, specifically typographic errors, were not affected by clinical interventions. This makes sense, as this type of error where, for example, two numbers in a patient MRN are transcribed incorrectly, is difficult to catch manually by humans, and is much better suited for an automated software platform such as the planning error tracking tool developed in this work. The failure of education to reduce this type of error means an automated solution is needed. Currently, our institution is looking at implementing DICOM Modality Worklist into our clinical workflow. This is a network connection that would be established between all of the CT simulators and record and verify systems in our Department and would allow for automatic transfer of patient demographics between systems. This would significantly decrease the amount of data that is manually entered by humans in our treatment planning process.

From our clinical experience, data errors caught in the late stages of the treatment planning process take significant time to fix, delaying the start time for patients. A time‐cost analysis was performed to estimate the impact of this software tool on the efficiency of generating treatment plans in our Department. In Table 3, we present the maximum time needed to correct each data error without the software implemented, which was used to calculate the time‐cost gains. In reality, there is a range in the time needed to correct any of the data error types. For example, if there is a slice missing in an image dataset, the time needed to fix this error could be only a few hours. This would be the case if the slice is located outside of the treatment volume entirely and is therefore clinically insignificant, or is located within the center of the target volume where recalculation of the dose distribution on the new CT does not significantly impact the dosimetric target coverage achieved. The scenario where a slice missing takes 1 day to fix is where that slice is located at the edge of the gross target volume for an IMRT plan. In this case, planning target volumes as well as associated optimization structures need to be regenerated and the case must then be reoptimized in order to ensure adequate target coverage. Similarly, for the data error where an incorrect image is in the secondary image list, the time needed to fix this error could be less than an hour if the physician can review the patient contours right away and the changes to contours are insignificant. However, if patient contours need to be changed, the case must then be replanned, and a day is needed to correct the error entirely. The time to correct a DICOM tag data error without the software was estimated to be 1 week in Table 3. This is based on the worst‐case scenario where the erroneous reference CT has been imported into the record and verify system, requiring a vendor support ticket be placed for removal. Technically, this situation could be fixed faster by exporting the reference CT directly from the treatment planning system to XVI, bypassing the record and verify system entirely. However, in our experience this method results in bypassing the numerical verification process of treatment isocenter information. Therefore, our institutional policy is to remove the erroneous CT from Mosaiq and then resend the DICOM CT data through the typical data export workflow for treatment plans (i.e., Pinnacle to Mosaiq to XVI).

Conservatively, using the time estimates in Table 3, the delays to patient start times in 2016 as a result of data errors without the software tool in place would have been 812 h vs 17.3 h with the tool in place. This significant time savings gained by using the planning error tracking tool comes from catching these types of data errors early in the treatment planning workflow. This minimizes the time needed to replan the case as well as remove erroneous data from the record and verify system. As a result of clinical interventions to our treatment planning workflow, the time cost for correcting data errors in 2017 was further reduced to 7.9 h with the software tool in place. Given that 6 of the 7.9 h needed to correct data errors in 2017 is due to manual data entry errors, the implementation of DICOM Modality Worklist is expected to reduce the total cost of data errors on treatment planning efficiency to less than 2 h.

One caveat to the times estimate analysis presented is that it excludes urgent cases where the entire plan is completed before the software is run in the evening. This was thought to be a reasonable exclusion for the analysis given that only 1 of the 121 data errors detected in 2016–2017 was associated with an urgent case. For this case, the treatment did not start until the next day, therefore, physics was able to fix the data error prior to treatment. This brings up a good point that with the software only being run in the evening, it is ineffective at catching data errors for urgent simulation and treatment cases. These are cases that one would expect to be at increased risk for data errors given that they are highly time sensitive. The reasoning for only running the software in the evening was to eliminate the impact of scanning the clinical databases on staff members trying to access them for treatment planning during the day. Given that a low number of urgent cases have had detected data errors thus far, running the software tool once daily still appears to be reasonable. Nevertheless, if the number of data errors associated with urgent cases were to increase, then additional scans by the software tool would be warranted despite its impact on the performance of the treatment planning system for users.

It should be mentioned that a limitation of this study is that the specific data errors discussed in this work, as well as the planning error tracking tool developed, are specific to the equipment used and treatment planning workflows practiced within our Department. Similarly, the time estimates to correct errors presented in this study are based on our user experience. Clinicians at other institutions may find that the magnitude of these errors, as well as the time to correct them, depends on the equipment they have as well as how their treatment planning workflow is designed. Despite this limitation, we feel that the work presented here highlights an area of the treatment planning process that has not been focused on in the past and for which errors can not only result in mistreatment of the patient but also significantly compromise clinical efficiency. This loss in efficiency is due to a lack of integrity in data communication processes between various software and hardware platforms in the treatment planning chain, an area for which the radiation oncology community is still gaining a better understanding. While users cannot use the software tool developed in this work and apply it directly to their own clinics, they can follow the framework outlined here to examine their own treatment planning process further and potentially develop their own data error tracking tool. Since the software developed is based on a C/C++ programming language, the programming tools needed to replicate this type of software are widely available. Getting access to the necessary databases to perform scanning may require users to work with their treatment planning and record and verify system vendors. If software development resources are not available, clinics could still use the framework presented here to track these types of data errors, and identify weaknesses in data integrity. Once identified, process improvements could be implemented to minimize data errors in their treatment planning workflow.

## 5 CONCLUSIONS

In summary, data errors occur in the treatment planning process and can significantly impact treatment plan creation, in terms of quality of the plans created, and delays in treatment start times for patients. This is especially true if errors are caught late in the treatment planning chain. In this work, we have successfully developed and utilized a software program aimed at detecting these data errors during the planning process. By automatically running this tool each night, patient files are checked at the early stages of treatment planning, thereby improving the accuracy and efficiency of plan generation at our center. A time‐cost analysis for the data errors detected in the first year of software implementation estimates a maximum potential time savings of 795 h that year. This is time that would otherwise have been spent fixing and replanning patient cases.

Use of this tool has identified weaknesses in our planning workflow that were not obvious by other quality assurance means and resulted in important clinical interventions. These interventions reduced the overall data error frequency by almost half, down to 42 occurrences in 2017 as compared to 79 occurrences in 2016. The data error type that was not impacted by clinical interventions implemented in 2017 was manual data entry errors. Therefore, our clinic is planning on changing the treatment planning workflow to minimize manual data entries by humans through use of DICOM Modality Worklist. Another important aspect of this software tool is the simplicity of its design and its ability to run without installing additional software on the treatment planning system servers. This allows us to easily and independently adapt the software as new errors are identified without vendor technical support. As such, this planning error tracking tool will continue to serve an important quality assurance role within our clinic.

## CONFLICT OF INTEREST

The authors have no other relevant conflicts of interest to disclose.
